# “Induced sputum versus gastric lavage for the diagnosis of pulmonary tuberculosis in children”

**DOI:** 10.1186/1471-2334-13-222

**Published:** 2013-05-16

**Authors:** Marta Ruiz Jiménez, Sara Guillén Martín, Luis M Prieto Tato, Juana B Cacho Calvo, Ana Álvarez García, Beatriz Soto Sánchez, Jose T Ramos Amador

**Affiliations:** 1 Department of Pediatrics, Getafe Hospital, Caterreta de Toledo Km 12.5, Madrid 28905, Spain; 2 Department of Microbiology, Getafe Hospital, Carretera de Toledo, Km 12.5, Madrid 28905, Spain

**Keywords:** Tuberculosis, Children, Induced sputum, Diagnosis

## Abstract

**Background:**

Diagnosis of pulmonary tuberculosis (PTB) is difficult in infants and young children. For microbiological confirmation of PTB children, sequential gastric lavage (GL) is recommended. Induced sputum (IS) may be an alternative or complementary tool, but the information is limited in children in developed countries. The aim of this study is to assess the safety and diagnostic yield from IS combined with GL for PTB diagnosis in non-HIV infected children.

**Methods:**

The study involved 22 children with suspected PTB admitted to the Getafe Hospital from January 2007 to May 2011. IS and GL were performed on three consecutive days, according to a standardized protocol. In all samples, BK staining, culture and PCR were carried out, including Genotype MTBDR plus for resistance to INH-RIF (Isoniazid-Rifampin) since 2008. A preliminary analysis of an ongoing prospective study is presented.

**Results:**

Median age was 72 months (range 1 month to 14 years of age). Seven (33%) were ≤ 5 years of age. Seventeen were clinically diagnosed of PTB based on positive PPD and radiological criteria. Microbiological confirmation was achieved in 10 (58.8%) by either GL or IS. *M. tuberculosis* was identified by GL in 8 children (47.1%) and by IS in 7 (41.2%). One infant (2 IS samples) had transient oxygen desaturation recovered spontaneously.

**Conclusions:**

IS appears to be safe and well tolerated by children for diagnosis of PTB and is more convenient. Increasing the diagnostic yield of PTB in children with PTB may be a complementary technique. Largest studies are necessary to define the role of IS in paediatric PTB.

## Background

Tuberculosis (TB) presents a high degree of variability of clinical manifestations that make diagnosis difficult. If this is the case with adults, it is even more complicated with children, where clinical and radiological signs can be non-specific and variable and there is greater difficulty in microbiological isolation. Microbiological confirmation of TB is desirable for definitive diagnosis. There is also a compelling need to obtain suitable samples that ensure culture growth in order to perform resistance studies. This is particularly important, given the emergence of multi-drugs and extensive drug-resistant strains. For microbiological confirmation of diagnoses of pulmonary tuberculosis (PTB) in young children, sequential gastric lavage (GL) is recommended. However GL is invasive, stressful, usually require the admission of children and overnight fasting. In the last years, there have been articles published in developing countries with a high prevalence of HIV-infection, in which induced sputum (IS) is used to diagnose TB in children. The technique is less invasive than GL, takes less time and can be performed on outpatients.

The IS yield in children with suspected TB has been variable, according to the different studies carried out in developing countries. There is very little information on the safety and sensitivity of IS in children in developed countries.

The aim of this study is to assess the safety and diagnostic yield of IS combined with GL for PTB diagnosis in non-HIV infected children.

Part of the study was presented at the 30th Annual Meeting of the European Society for Paediatric Infectious Diseases [1].

## Methods

A prospective pilot study was performed at the Department of Paediatrics at the Getafe Hospital, a tertiary university hospital that covers a population of around 33,000 children under 15 years of age. The study served as the predecessor of a large prospective multi-centre study that is currently underway at 21 hospitals in Madrid.

Children enrolled in the study were consecutive patients admitted to hospital from January 2007 to May 2011 with suspected PTB. Inclusion criteria were clinical or radiological suspicion of PTB and no evidence of immunosuppression.

Children were considered to be of immigrant origin if they were born to foreign parents, either abroad or in Spain.

PTB was diagnosed according to clinical or epidemiological data.

Prior to enrolment of a patient in the study, informed consent was obtained from parents or legal guardians. Clinical research ethics committee of Getafe Hospital (reference number 12/61) approved the protocol as part of a multi-centre study conducted in Madrid. Medical history was recorded and a physical examination performed on every child enrolled. At our hospital, HIV testing is not routinely carried out on children with suspected PTB. It was performed only on children from areas with a high prevalence of HIV.

IS and GL were performed on three consecutive days, according to a standardized protocol. In all samples, BK staining, culture and PCR were carried out, including Genotype MTBDR plus for resistance to INH-RIF since December 2008. Specimens were sent to the laboratory immediately after being obtained.

GL was performed early morning on all children, after an overnight fast. A nasogastric tube was passed and normal saline 20 ml inserted, left for 3 minutes and then aspirated. An additional 5–10 ml of normal saline was introduced and aspirated, until a minimum of 20 ml of aspirate was obtained.

IS was performed 4 hours after GL, prior to lunch. To prevent the risk of bronchospasm induced by hypertonic saline, children were pre-treated with nebulized salbutamol (0.03 ml/kg, maximum 1 ml (1 ml = 5 mg)). Subsequently, 5 ml of 5% sterile saline at a flow rate of 5 L per minute was nebulized for 15 minutes, followed by chest percussion on the front and back chest wall. After this procedure, if spontaneous expectoration was not achieved, sputum was obtained by suctioning through the nasopharynx with a sterile mucus extractor of catheter size 6 or 7. Oxygen saturation was monitored throughout the IS. After the procedure, a doctor performed a physical examination to rule out the presence of bronchospasm.

Clinical specimens were processed as follows: the specimens were firstly digested and decontaminated by the N-acetyl-L-Cysteine-NaOH method (BioMérieux, Spain). After decontamination, the concentrated sediment was suspended in 2 ml sterile phosphate buffer (pH 7.0) and auramine-rhodamine acid fast staining was performed. An aliquot of the decontaminated specimens was cultured on a Coletsos solid medium and Bactec MGIT 960 liquid medium (Becton Dickinson, Maryland USA). After inoculation for growth detection, the remaining decontaminated specimen was stored at −20°C for use in Nucleic-acid amplification tests (NAAT). The identification of *M. tuberculosis complex* in cultures with growth was confirmed by AccuProbe (GenProbe, Inc, San Diego, CA) and GenoType® MTBDRplus assay (Hain Lifescience, Nehren, Germany) before and after December 2008 respectively. One strain from every patient from which *M. tuberculosis* was isolated was kept at −20°C.

Commercial Nucleic-acid amplification tests (NAATs) used for the study period were COBAS TaqMan MTB (Roche Molecular Systems, Basel, Switzerland), GenoType® MTBDRplus assay (Hain Lifescience, Nehren, Germany) and Xpert® MTB/RIF (Cepheid, Sunnyvale, CA). They were introduced step by step into the laboratory procedures.

A *Mycobacterium tuberculosis Complex* strain from each patient was tested by a drug susceptibility test (DST). Testing for susceptibility to commonly used drugs including isoniazid (INH), rifampin (RMP), streptomycin (S), ethambutol (E) and pyrazinamide (PZA) on TB-positive culture was performed by the BACTECTM MGIT 960 system (Becton Dickinson, Maryland USA) following the manufacturer’s protocol.

### Statistical analysis

Statistical analysis was performed with the program SPSS Statistics Base 17.0, using the two-sided McNemar test to compare the yield of *M. tuberculosis* from EI and GL.

## Results

Twenty-two patients were admitted with suspected PTB. One was excluded for not having provided the collection of samples according to the protocol. 11 patients (53%) were females and the median age was 72 months (range 1 month to 14 years of age). Seven (33%) were ≤ 5 years of age. 9 patients (43%) were from Spain and 12 (57%) from other countries: South America (7), Africa (4) and Eastern Europe (Ukraine) (1).

Seventeen patients (80%) were clinically diagnosed of PTB, based on a positive tuberculin skin test and radiological criteria. Four patients were excluded because the final diagnosis was not PTB and other microorganisms were identified. In all of them, it was carried out the same diagnostic tests (3 GL and 3 IE). Follow up of the children were satisfactory without antituberculous drugs.

A positive tuberculin skin test was considered if the Mantoux reaction was > 10 mm, regardless of the existence of BCG vaccination history or >5 mm and exposure was to a well-known TB source. Of these patients, the reason for study was: 8 (47%) TB exposure (3 patients asymptomatic), one (5.9%) because of screening of children from an endemic area and 8 (47%) the presence of symptoms. At the time of diagnosis, 11 (65%) patients had clinical symptoms, the most common, respiratory symptoms and fever 5 (29.4%), respiratory symptoms isolated 4 (23.5%) and constitutional symptoms 2 (11.8%). In all patients, the Mantoux was greater than 5 mm and the median 18.4 mm (range 8–20). In one patient of two months old, the tuberculin skin test became positive during the follow-up by tuberculosis contact with initially normal chest radiographs, which subsequently became pathological. Six patients were born abroad. In 5 of them the Mantoux was above 10 mm. In another patient, the Mantoux was 8 mm, but with pathological chest radiography and microbiological isolation of *M. tuberculosis*.

Chest radiographs were performed in all cases: pathological in 12 (70%) patients (lymphadenopathy being the most common finding), normal in 4 (23.5%) patients and uncertain in 1 (5.9%). In the 5 patients with normal or uncertain chest radiographs, thoracic CT was performed and pathological in all. The index case was known in 9 (53%) patients: father (1), mother (2), grandfather (2) and uncle (4).

An HIV-test (enzyme-linked immunoassay, ELISA) was performed on only one patient, with negative results. From rest of the patients, family history of all household contacts, including the mother, was collected and none were HIV-infected. Since vertical transmission is the mayor route of HIV-transmission we assumed that they were not HIV-infected. No other risk factors were identified.

Interferon gamma assay (QuantiFERON-TB Gold test) was performed with 4 patients (23.5%): 2 positive, 1 negative and 1 undetermined.

In all patients included in the study (21), IS was successfully performed. No patient was too ill to undergo the technique. The youngest patient was a 34-day old baby girl, who did not show any adverse effects. A 9-year old patient with pleural effusion showed no undesirable effects. Only two patients of 13 and 14 years of age respectively expectorated spontaneously, without aspiration.

In total, there were 63 IS samples. In the first 5 patients (January 2007 - June 2008) Secondary effects were not recorded, as the data was not prospectively gathered until June 2008. Of the 16 patients that showed potential adverse IS effects (a total of 48 procedures), no serious adverse reactions occurred during or after the procedure; the most common adverse events were mild epistaxis in 8 procedures (16.6%), nausea in 3 (6.25%) and increased coughing in 3 (6.25%). Only one infant had transient hypoxemia in 2 procedures (lowest oxygen saturation 87%) recovered spontaneously. There were no episodes of bronchospasm.

Microbiological confirmation was recorded in 10 patients (58.8%) by either GL or IS. *M. tuberculosis* was identified from GL in 8 children (47.1%) and by IS in 7 (41.2%) (Additional file 1: Figure and Table S1).

In Table 1 are reflected microbiological results of the 10 patients with microbiological confirmation. Five children were positive on both samples collection methods. In two patients, microbiological diagnosis was possible only by IS (PCR) and in three patients only by GL. The coefficient of correlation (Kappa index) for the GL and IS was 0.401.

**Table 1:** Patients with positive result in GL and/or IE

**Patient**	**Induced sputum**			**Gastric lavage**		
	**Culture positive**	**Smear positive**	**PCR positive**	**Culture positive**	**Smear positive**	**PCR positive**
1	3 (3)	1 (3)	NP	2 (3)	0	NP
2	1 (3)	0	0	3 (3)	0	0
3	3 (3)	0	0	2 (3)	0	0
4	3 (3)	1 (3)	3 (3)	3 (3)	3 (3)	3 (3)
5	3 (3)	0	0	3 (3)	0	1 (3)
6	0	0	0	1 (3)	0	0
7	0	0	0	1 (3)	0	0
8	0	0	0	2 (3)	0	0
9	0	0	1 (3)	0	0	0
10	0	0	1 (3)	0	0	0

NP: not performed.

Of the total IS samples (51) from 17 patients diagnosed with TB, the culture was positive in 13 (25.5%) and smear in 2 (3.9%). Of the 51 samples from gastric aspirate, the culture was positive in 17 (33.3%) and smear in 3 (5.9%). Thirty-five clinical samples (17 IS and 18 GL) from 13 patients were processed by NAAT. Twenty-three samples (12 IS and 11 GL) were processed by COBAS TaqMan MTB, 11 (4 IS and 7 GL) by GenoType ® MTBDRplus and one IS sample by Xpert® MTB/RIF. NAAT was positive in 5 of the 17 IS samples (29.4%) and in 3 of 18 GL samples (16.7%) (Additional file 1: Table S1).

Table 2 shows the cumulative yields from IS and GL, after taking successive samples on 3 consecutive days and the combined yield of both techniques.

**Table 2:** Cumulative yields from GL and IS and combined yield of both techniques

	**Patients**	**Cumulative yield**	**95% IC**
**Induced sputum**			
**1st day**	17	5/17 (29.4%)	29.4–56%
**1st + 2nd day**	17	6/17 (35.3%)	35.3–61.7%
**1sº + 2nd + 3rd day**	17	7/17 (41.2%)	18.4–67.1%
**Gastric lavage**			
**1st day**	17	7/17 (41.2%)	18.4–67.1%
**1st + 2nd day**	17	7/17 (41.2%)	18.4–67.1%
**1st +2nd + 3rd day**	17	8/17 (47.1%)	23–72%
**Induced sputum + Gastric lavage**		**Combined yields**	
**1st day**	17	8/17 (47.1%)	23–72%
**1st + 2nd day**	17	9/17 (53%)	52.9–77%
**1st + 2nd + 3rd day**	17	10/17 (58.8%)	32.9–81.6%

The diagnostic yield from three sequential GL was 8/17 (47.1%), equivalent to that of one sample from GL and one sample from IS on the same day (8/17) (47.1%).

There was no significantly statistical difference between the diagnostic yields of three GL samples and three IS samples (p 0.81), nor between three GL samples and the combination of both techniques on three consecutive days (p 1).

Of the seventeen patients diagnosed of PTB, 5 were ≤ 5 years of age (ages: 2, 6, 8, 28 and 47 months). All of these patients had microbiological confirmation, except in the case of the 28 months-old child. In two cases (2 months-old and 8 months-old infants) both samples collection methods isolated the *M.tuberculosis*.

There were two cases of resistance (one to isoniazid in a Spanish child and the other to rifampicin in an immigrant child). The case of rifampicin resistance was detected in culture from GL (where the microbiological results of all samples from IS were negative). The case of resistance to isoniazid was detected in culture samples of GL. In this patient, PCR and culture in the three IS samples were also positive. Resistance study was initially perform into the 1st IS (PCR), Genotype MTBDR plus was negative. This, following the outcome of GL culture, was interpreted as a discrepancy of results. No resistance study was conducted on samples of IS culture, having been already performed in the culture from GL.

## Discussion

In recent years, there has been a worldwide increase of cases of drug-resistant and multidrug-resistant tuberculosis. In Spain, there has also been an increase in the rate of resistance in the general population [2]. Children are no exception to the problem and show the same rate as adults. Priority is therefore to optimize diagnosis, especially in cases in which the index case is not known. In our study, the cumulative yield of GL on three consecutive days (47.1%) and cumulative yields from IS on three consecutive days (41.2%) is lower than the combined yield from both techniques (58.5%). IS could be a complementary technique to increase the diagnostic yield of PTB in children.

We have shown that the diagnostic yield from three sequential GL was equivalent to that from one GL sample and one sample from IS on the same day. The realization of both techniques on one day may decrease the length of stay and cost, as well as reduce inconvenience to families. It could be an alternative in regions with limited health care resources [3].

Isolation rates for *M tuberculosis* from GL ranged from 28% to 40% in children with suspected PTB, although rates can rise to 75% in other series [4-7]_._ In our series, the diagnostic yield from GL was 47.1% and IS 41.1%. This relatively high percentage of isolates in both techniques may be due to the use of a standardized technique and the rapid laboratory processing of specimens. The amount of time that elapses between sample collection and the start of a microbiological study is a determining factor for obtaining positive results.

The diagnostic yield from IS was 41.1%. In previous studies, where IS was used in the diagnosis of TB in children, the results were variable. Shata et al. published a prospective study on 30 children between 3 and 15 years of age, in which the microbiological isolation from IE (staining or culture) was 28%. However, GL was not measured [8]. Zar et al., in two different studies, recorded 10% and 22% respectively [9,10]. The first prospective study included 149 children in South Africa, with ages ranging from 3–20 months, hospitalized for pneumonia with a high risk of PTB. Only one sample from IS was compared to one to three from GL [9]. The second was a prospective study on 250 children admitted for suspected PTB. All patients underwent three ISs and three GLs (on consecutive days) [10]. Iriso et al. carried out a study on a total of 126 children aged 2–60 months, recruited as probable TB cases. The diagnostic yield from IS was 30% [11]. In the study carried out by Hatherill et al., the diagnostic yield from EI was 5.8% [3]. These differences may be due to the fact that not all studies performed 3 measurements of IS and to the characteristics of the populations included in the studies, with different degrees of risk of tuberculosis. Zar et al. in the first study included patients admitted for pneumonia [9]. Hatherill et al. performed the study on outpatients with tuberculosis contact or symptoms consistent with TB [3]. In our study, the high isolation rate achieved by IS may be due to the fact that 3 samples were obtained, including by PCR performed on a study population with a high likelihood of PTB.

A study conducted at a Madrid hospital was recently carried out to compare IS and GL in the diagnosis of PTB in children. Both techniques were performed on 26% patients diagnosed with PTB. Microbiological diagnosis was performed by GL in 8 patients (30.8%) and IS in 2 (7.7%). Of the total of 77 GL samples, the culture was positive in 19 (24.77%), whereas from the 75 IS samples, the culture was positive only in 3 (4%), with a statistical significance (p = 0.03, odds ratio 1.33 (95% CI 0.89-1.98). The diagnostic GL yield GL exceeded that of IS by more than 20%. The authors believe that this difference from the literature may be due to the variability of the sampling process and the greater difficulty in obtaining a valid IS [12].

The rate of smear-positive cases was low in both GL and IS, being lower in IS. This contrasts with other studies, in which the smear-positive in IS ranged between 9 and 12%, higher than is usually achieved in GL [8,10,11].

The fact that in two children under 1 year of age IS was positive, suggests that IS can be achieved good results in younger children.

IS is relatively simple and does not require sophisticated equipment. However, it has the disadvantage of an increased risk of transmission, since with the IS, Flugge droplets are produced. The risk of transmission of TB with this technique is lower in children than in adults, due to the paucibacillary nature of the disease at a lower age. Nevertheless, it is essential to adopt strict environmental control measures to prevent nosocomial transmission to other patients or health-care staff. It is recommended that the technique be performed in a room with negative pressure [13,14]. If this system is not available, the technique must be performed in a room with adequate ventilation. In addition, personal protection measures should be taken. All health-care workers in contact with patients diagnosed or suspected of having TB, must use masks with HEPA filters [14,15].

In studies carried out on adults, there have been adverse effects of induced sputum, transient hypoxemia, bronchospasm or increased volume of pre-existing pleural effusion, the risk being higher in moderately or severely ill patients and HIV infected patients [16,17]. In our study, as in other studies on children, induced sputum was well-tolerated [3,8-10]. No serious adverse reactions occurred during or after the procedure, even in one child with pleural effusion. The youngest patient was a 34-day old baby girl, who did not show any adverse effects. Unlike other studies on children, in our study population, there were no patients HIV infected or severely ill [3,9-11].

Several potential study limitations should be taken into account. Firstly, the small sample size does not allow for conclusive results. The presence of polymorphonuclear cells in the specimen is indicative of sputum, in contrast to epithelial cells, which originate in saliva. The sputum was not examined to determine whether polymorphonuclear or epithelial cells, predominated. The validity of our results may not be extrapolated to other settings. The prevalence of TB and HIV-infection may be major determinants of diagnostic yield.

Nevertheless, our study is one of the first prospective studies carried out on children in developed countries with a low prevalence of HIV-infection. This initial data from the pilot study warrants more extensive studies.

## Conclusion

IS is a safe and well- tolerated technique and can be successfully performed even on infants. In children with suspected PTB, GL remains the standard technique for microbiological diagnosis. However, taking in account the increasing problem of resistant strains, IS should also be considered at least in developing countries, as a complementary tool to increase the diagnostic yield of PTB. Largest studies are necessary to define the role of IS in paediatric PTB.

## Competing interest

The authors don´t have any commercial or other associations that might pose a conflict of interest.

## Authors’ contributions

RJM devised and supervised the study, analysed the results and was mainly responsible for writing the paper. GMS, PTLM, AGA and SSB enrolled patients and gathered data. CCJB was responsible for the microbiological issues. RAJT took part in the design of the protocol, analysed results and the paper. All authors read and approved the final manuscript.

## Review board

Maria Jose Mellado, Sofia Samper, Beate Kampmann and Jacobus de Waard.

## Pre-publication history

The pre-publication history for this paper can be accessed here:

http://www.biomedcentral.com/1471-2334/13/222/prepub

## Supplementary Material

Additional file 1: Figure S1 and Table S1 Cumulative yield of *M. tuberculosis* from repeated induced sputum (IS) or gastric lavage (GL) specimens. Click here for file
