# Supplementary material for: “Induced sputum versus gastric lavage for the diagnosis of pulmonary tuberculosis in children”
Source: BMC Infect Dis. 2013 May 16;13:222. doi: 10.1186/1471-2334-13-222 (PMC3688294; doi:10.1186/1471-2334-13-222)
Supplement: Additional file 1: Figure S1 and Table S1 — Cumulative yield of M. tuberculosis from repeated induced sputum (IS) or gastric lavage (GL) specimens. [file 1471-2334-13-222-S1.docx]

**Figure 1 and Tabla 1. Cumulative yield of *M. tuberculosis* from repeated induced sputum (IS) or gastric lavage (GL) specimens.**

**Percentage**

**Smear positive**

**PCR positive**

**Culture positive**

**Cumulative yield**

**IS**

**IS**

**GL**

**IS**

**IS**

**GL**

**GL**

**GL**

Note: Solid columns indicate the induced sputum (IS) group and dotted columns indicate the gastric lavage (GL) group.

|  | **Patients** | **Smear positive** | **PCR positive** | **Culture positive** | **Cumulative yield** |
| --- | --- | --- | --- | --- | --- |
| **Total** | 17 | 2 (11.8%) | 4 (23.5%) | 8 (47%) | 10 (58.8%) |
| **Induced sputum** | | | | | |
| **Total**  **1º specimen**  **2º specimen**  **3º specimen** | 17  17  17  17 | 2 (11.8%)  1 (5.9%)  0 (0%)  1 (5.9%) | 3 (17.6%)  2 (11.8%)  2 (11.8%)  1 (5.9%) | 5 (29.4%)  4 (23.5%)  4 (23.5%)  5 (29.4%) | 7 (41.2%)  5 (29.4%)  5 (29.4%)  5 (29.4%) |
| **Gastric lavage** | | | | | |
| **Total**  **1º specimen**  **2º specimen**  **3º specimen** | 17  17  17  17 | 1 (5.9%)  1 (5.9%)  1 (5.9%)  1 (5.9%) | 2 (11.8%)  1 (5.9%)  0 (0%)  2 (11.8%) | 8 (47%)  7 (41.2%)  5 (29.4%)  5 (29.4%) | 8 (47.1%)  7 (41.2%)  5 (29.4%)  5 (29.4%) |
